# Molecular mechanism of wedelolactone inhibits high glucose-induced human retinal vascular endothelial cells injury through regulating miR-190 expression

**DOI:** 10.1097/MD.0000000000037388

**Published:** 2024-05-24

**Authors:** Xiaojie Cai, Xiao Wang, Yuping Huang, Xiaopang Rao

**Affiliations:** a Department of Nursing, People’s Hospital of Chengyang District, Qingdao City, Shandong Province, China; b Department of Ultrasound, People’s Hospital of Chengyang District, Qingdao City, Shandong Province, China; c Center of Health Management, People’s Hospital of Chengyang District, Qingdao City, Shandong Province, China

**Keywords:** apoptosis, miR-190, oxidative stress, retinal vascular endothelial cells, wedelolactone

## Abstract

To investigate the effects and molecular mechanisms of wedelolactone (WEL) on high glucose-induced injury of human retinal vascular endothelial cells (HRECs). The cell injury model was established by incubating HRECs with 30 mmol/L glucose for 24 hour. HRECs were divided into control (Con) group, high glucose (HG) group, HG + WEL-low dose (L) group, HG + WEL-medium dose (M), HG + WEL-high dose (H) group, HG + miR-NC group, HG + miR-190 group, HG + WEL + antimiR-NC group, HG + WEL + antimiR-190 group. The kit detects cellular reactive oxygen species (ROS), superoxide dismutase (SOD), and malondialdehyde (MDA) content; cell apoptosis was analyzed by flow cytometry; miR-190 expression was detected by real-time quantitative PCR (RT-qPCR). Compared with Con group, the levels of ROS and MDA in the HG group were significantly increased (*P* < .01), the SOD activity and the expression of miR-190 expression were significantly decreased (*P *< .05), and the apoptosis rate was significantly increased (*P* < .01). Compared with HG group, the levels of ROS and MDA in HG + WEL-L group, HG + WEL-M group and HG + WEL-H group were significantly decreased (*P *< .05), SOD activity and miR-190 expression were significantly increased (*P* < .05), and apoptosis rate was significantly reduced (*P *< .05). Compared with the HG + miR-NC group, the levels of ROS and MDA in HG + miR-190 group were significantly reduced (*P* < .01), SOD activity was significantly increased (*P* < .01), and apoptosis rate was significantly reduced (*P *< .05). Compared with the HG + WEL + antimiR-NC group, the ROS level and MDA content in the HG + WEL + antimiR-190 group were significantly increased (*P *< .05), SOD activity was significantly decreased (*P *< .05), and apoptosis rate was significantly increased (*P *< .05). Wedelolactone can attenuate high glucose-induced HRECs apoptosis and oxidative stress by up-regulating miR-190 expression.

## 1. Introduction

Diabetic retinopathy (DR) is one of the major complications of diabetes and is the leading cause of acquired blindness in working-age adults.^[1]^ High blood glucose is a major triggering factor for the progression of DR, as it promotes oxidative stress and induces damage to human retinal endothelial cells (HRECs).^[2]^ Finding effective treatments for DR is of utmost importance.

Wedelolactone (WEL) extract, derived from the aerial parts of the Asteraceae plant *Eclipta prostrata L*., has been shown to possess pharmacological properties such as antioxidant, antitumor, hepatoprotective, and anti-inflammatory effects.^[3–5]^ Previous studies have demonstrated that WEL extract can inhibit oxidative stress response and alleviate hydrogen peroxide-induced oxidative stress damage in vascular endothelial cells.^[6,7]^ However, the protective effects and mechanisms of WEL on high glucose-induced damage in HRECs have not been reported.

MicroRNAs (miRNAs) are endogenous noncoding RNAs of approximately 20 to 23 nucleotides in length, and their altered expression is involved in key pathways such as oxidative stress, inflammation, retinal neurodegeneration, and autophagy in the pathogenesis of DR.^[8]^ It has been found that miR-190 is downregulated in hydrogen peroxide-treated H9c2 cardiomyocytes, and overexpression of miR-190 can protect H9c2 cells from apoptosis and oxidative stress damage induced by hydrogen peroxide.^[9]^ However, there is limited research on the protective effects of miR-190 on high glucose-induced damage in HRECs.

In this study, we aimed to investigate the effects of WEL extract on high glucose-induced damage in HRECs and explore the potential involvement of miR-190 as a mechanism. Our findings will provide experimental evidence for the use of WEL in the treatment of Dr

## 2. Materials and methods

### 2.1. Materials

All experimental tests were conducted at the Research Laboratory of Ocean University of China, funded by the Key Discipline Fund. HRECs were purchased from the Cell Bank of the Shanghai Institutes for Biological Sciences, Chinese Academy of Sciences. WEL (batch number 111885-201804, purity 95.0%) was obtained from the China Institute for Food and Drug Control. Lipofectamine 2000 was purchased from Invitrogen Corporation (Waltham, USA). miR-190 mimics, antimiR-190, and their respective controls (miR-NC, antimiR-NC) were provided by Beijing Liuhe Huada Gene Company. Reactive oxygen species (ROS) detection kit, Annexin-V-FITC/propidium iodide (PI) apoptosis detection kit, and ECL staining kit were purchased from Shanghai Biyun Tian Biological Company (Shanghai). Malondialdehyde (MDA) content detection kit and superoxide dismutase (SOD) activity detection kit were obtained from Beijing Solaibao Biological Company (Beijing). Cleaved-caspase3 rabbit polyclonal antibody (ab2302), cleaved-caspase9 rabbit polyclonal antibody (ab2324), and glyceraldehyde-3-phosphate dehydrogenase (GAPDH) rabbit polyclonal antibody (ab9485) were purchased from Abcam (Cambridge, USA).

### 2.2. Methods

#### 2.2.1. Cell culture, transfection, and experimental groups.

HRECs were seeded in low-glucose DMEM medium containing 10% fetal bovine serum (glucose concentration of 5.5 mmol/L) and cultured in a 5% CO_2_, 37°C incubator. When the cells reached 80% confluency, they were digested with trypsin and passaged at a 1:3 ratio. Log-phase HRECs were seeded into 6-well plates at a density of 2 × 105 cells per well. According to the instructions of Lipofectamine 2000, HRECs were transfected with miR-190 mimics, miR-NC, antimiR-190, and antimiR-NC, respectively, in 50% confluent cells. The transfected cells were collected after 48 hours for subsequent experiments.

Experimental groups were as follows:

1. Control (Con) group: HRECs cultured in medium containing 5.5 mmol/L glucose.
2. High glucose (HG) group: HRECs cultured in medium containing 30 mmol/L glucose [9].
3. HG + WEL-low dose (L) group, HG + WEL-medium dose (M) group, HG + WEL-high dose (H) group: HRECs cultured in medium containing 10, 20, and 40 μmol/L WEL^[6]^ and 30 mmol/L glucose. HG + miR-NC group, HG + miR-190 group: HRECs transfected with miR-NC or miR-190 mimics and cultured in the concentration of 5.5 mmol/L glucose. HG + WEL + antimiR-NC group, HG + WEL + antimiR-190 group: HRECs transfected with antimiR-NC or antimiR-190 and cultured in medium containing 40 μmol/L WEL and 30 mmol/L glucose. After 24 hours of culture, the cells were digested with trypsin for subsequent analysis.

The ethical approval was not necessary, since the research primarily involves cell experiment.

#### 2.2.2. Measurement of ROS levels in cells using a detection kit.

DCFH-DA was diluted in serum-free culture medium at a 1:1000 ratio to achieve a final concentration of 10 μmol/L. After collection, HRECs were suspended in the diluted DCFH-DA solution at a cell concentration of 1 × 106 cells/mL and incubated at 37°C with gentle inversion every 3 to 5 minutes for 20 minutes. The cells were then washed 3 times with serum-free culture medium to remove the excess DCFH-DA that did not enter the cells. The total fluorescence intensity of the cells was measured using a flow cytometer, with a positive control sample used to determine the level of ROS production.

#### 2.2.3. Measurement of SOD activity and MDA content in cells using detection kits.

HRECs were collected and centrifuged, and the pellet was resuspended in an extraction buffer. The cells were then sonicated to disrupt the cells, and the supernatant was collected. The SOD activity and MDA content in HRECs were measured according to the instructions of the SOD activity detection kit and MDA content detection kit, respectively.

#### 2.2.4. Detection of apoptosis rate using flow cytometry.

HRECs were adjusted to a concentration of 1 × 106 cells/mL using 1 × binding buffer. About 5 µL of Annexin-V-FITC and 5 µL of PI were added to 500 µL of cell suspension and stained in the dark for 20 minutes. The fluorescence signals were collected using a flow cytometer, and the apoptosis rate of the cells was analyzed using FlowJo 8.7.1 software.

#### 2.2.5. Detection of cleaved-caspase3 and cleaved-caspase9 protein expression using western blot.

HRECs from each group were washed once with PBS and lysed in radioimmunoprecipitation assay buffer on ice. Forty micrograms of protein were loaded per lane, separated by sodium dodecyl sulfate-polyacrylamide gel electrophoresis, and transferred onto a polyvinylidene fluoride membrane. The membrane was incubated with blocking buffer at room temperature for 5 hours, followed by overnight incubation at 4°C with primary antibodies against cleaved-caspase3 (1:200), cleaved-caspase9 (1 µg/mL), and the internal control GAPDH (1:2500). The membrane was then incubated with the corresponding secondary antibodies at room temperature for 1 hour. Immunobands were detected using an ECL staining kit, and the relative grayscale values of the bands were determined using Image-pro Plus software.

#### 2.2.6. Detection of miR-190 expression using real-time quantitative PCR (RT-qPCR).

Total RNA was extracted from HRECs using TRIzol reagent. About 2 µg of total RNA were reverse transcribed into cDNA using a miRNA reverse transcription kit. RT-qPCR was performed using miR-190 primers, U6 primers as internal reference, and a miRNA fluorescence quantitative kit. The upstream primer for miR-190 was 5′-GGTCTTTGATGATGATTCTGG-3′, the downstream primer was 5′-CTAGGCACAGTATTGAAGGTT-3′; the upstream primer for U6 was 5′-GCGCGTCGTGAAGCGTTC-3′, and the downstream primer was 5′-GTGCAGGGTCCGAGGT-3′. The expression level of miR-190 was calculated using the 2^−△△Ct^ method.

### 2.3. Statistical analysis

Three replicate wells were set up for each group, and the experiments were independently repeated 3 times. The experimental data were expressed as mean ± standard deviation (mean ± SD) if they followed a normal distribution and had equal variances. Independent sample *t* test was used for comparisons between 2 groups, and one-way analysis of variance (ANOVA) followed by SNK-q test was used for comparisons among multiple groups. A *P* value of <.01 was considered statistically significant.

## 3. Results

### 3.1. The effects of WEL on oxidative stress in high glucose-induced HRECs

Compared to the control group (Con), the high glucose group (HG) showed a significant decrease in SOD activity (*P* < .01) and a significant increase in MDA content and ROS levels (*P* < .01). Compared to the HG group, the HG + WEL-L, HG + WEL-M, and HG + WEL-H groups showed a significant increase in SOD activity (*P* < .01) and a significant decrease in MDA content and ROS levels (*P* < .01). There were statistically significant differences in SOD activity, ROS levels, and MDA content among the HG + WEL-L, HG + WEL-M, and HG + WEL-H groups (Table 1).

**Table 1:** The effects of wedelolactone on high glucose-induced oxidative stress in HRECs. (mean ± SD, *n* = 9).

Groups	ROS (%)	SOD (U/mg)	MDA (nmol/mg)
Con	100.00 ± 0.00	127.70 ± 11.52	3.15 ± 0.37
HG	340.74 ± 20.77*	34.86 ± 3.52*	19.96 ± 1.58*
HG + WEL-L	265.01 ± 21.56†	61.52 ± 5.08†	14.98 ± 1.12†
HG + WEL-M	203.85 ± 16.55†‡	81.62 ± 6.36†‡	10.15 ± 0.87†‡
HG + WEL-H	136.91 ± 14.25†‡§	101.79 ± 10.33†‡§	5.21 ± 0.48†‡§
*F*	308.830	181.117	441.948
*P*	<0.01	<0.01	<0.01

Compared to the Con group

* *P* < .05; compared to the HG group,

† *P* < .05; compared to the HG + WEL-L group,

‡ *P* < .05; compared to the HG + WEL-M group,

§ *P* < .05

### 3.2. The effect of WEL on apoptosis in high glucose-induced HRECs

**Figure 1.**
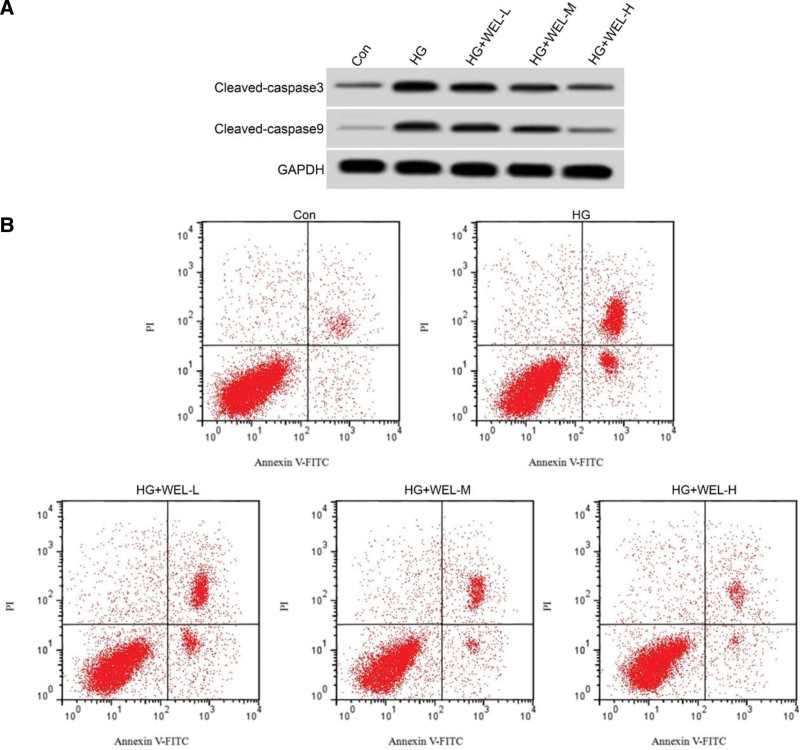
Effects of Wedelactone on apoptosis of HRECs induced by high glucose. HRECs. human retinal vascular endothelial cells. Con = control group, HG = high glucose group.

Compared to the control group, the HG group showed a significant increase in apoptosis rate, cleaved-caspase3, and cleaved-caspase9 protein expression (*P* < .05). Compared to the HG group, the HG + WEL-L, HG + WEL-M, and HG + WEL-H groups showed a significant decrease in apoptosis rate, cleaved-caspase3, and cleaved-caspase9 protein expression (*P* < .05). There were statistically significant differences in apoptosis rate, cleaved-caspase3, and cleaved-caspase 9 protein expression among the HG + WEL-L, HG + WEL-M, and HG + WEL-H groups (Figure 1; Table 2).

**Table 2:** Effects of wedelactone on apoptosis of HRECs induced by high glucose (mean ± SD, *n* = 9).

Groups	Apoptosis (%)	Cleaved-caspase 3	Cleaved-caspase9
Con	6.83 ± 0.59	0.22 ± 0.02	0.12 ± 0.02
HG	31.67 ± 3.02*	0.71 ± 0.06*	0.58 ± 0.04*
HG + WEL-L	24.22 ± 2.16†	0.57 ± 0.04†	0.41 ± 0.03†
HG + WEL-M	17.17 ± 1.19†‡	0.43 ± 0.04†‡	0.29 ± 0.03†‡
HG + WEL-H	9.89 ± 0.86†‡§	0.31 ± 0.03†‡§	0.17 ± 0.02†‡§
*F*	287.833	215.111	372.107
*P*	<0.01	<0.01	<0.01

Compared to the Con group

* *P* < .05; compared to the HG group,

† *P* < .05; compared to the HG + WEL-L group,

‡ *P* < .05; compared to the HG + WEL-M group,

§ *P* < .05

### 3.3. The effect of WEL on miR-190 expression in high glucose-induced HRECs

Compared to the control group, the HG group showed a significant decrease in miR-190 expression in HRECs (*P* < .01). Compared to the HG group, the HG + WEL-L, HG + WEL-M, and HG + WEL-H groups showed a significant increase in miR-190 expression in HRECs (*P* < .01). There were statistically significant differences in miR-190 expression among the HG + WEL-L, HG + WEL-M, and HG + WEL-H groups (Table 3).

**Table 3:** The effect of Wedelolactone on miR-190 expression in high glucose-induced HRECs (mean ± SD, *n* = 9).

Groups	miR-190
Con	1.00 ± 0.00
HG	0.28 ± 0.03*
HG + WEL-L	0.42 ± 0.04†
HG + WEL-M	0.59 ± 0.04†‡
HG + WEL-H	0.72 ± 0.06†‡§
*F*	451.870
*P*	<0.05

Compared to the Con group

* *P* < .05; compared to the HG group,

† *P* < .05; compared to the HG + WEL-L group,

‡ *P* < .05; compared to the HG + WEL-M group,

§ *P* < .05

### 3.4. The effect of miR-190 overexpression on oxidative stress in high glucose-induced HRECs

Compared to the HG + miR-NC group, the HG + miR-190 group showed a significant increase in miR-190 expression and SOD activity in HRECs (*P* < .01), and a significant decrease in ROS levels and MDA content (*P* < .01) (Table 4).

**Table 4:** The effect of miR-190 overexpression on oxidative stress in high glucose-induced HRECs (mean ± SD, *n* = 9).

Groups	miR-190	ROS (%)	SOD (U/mg)	MDA (nmol/mg)
HG + miR-NC	1.00 ± 0.00	344.30 ± 25.63	36.61 ± 3.19	20.49 ± 1.73
HG + miR-190	3.31 ± 0.22*	157.45 ± 14.36*	87.91 ± 8.54*	7.15 ± 0.64*
*t*	31.500	19.080	16.882	21.696
*P*	<0.05	<0.05	<0.05	<0.05

Compared to HG + miR-NC group,

* *P* < .05

### 3.5. The effect of miR-190 overexpression on apoptosis in high glucose-induced HRECs

**Figure 2.**
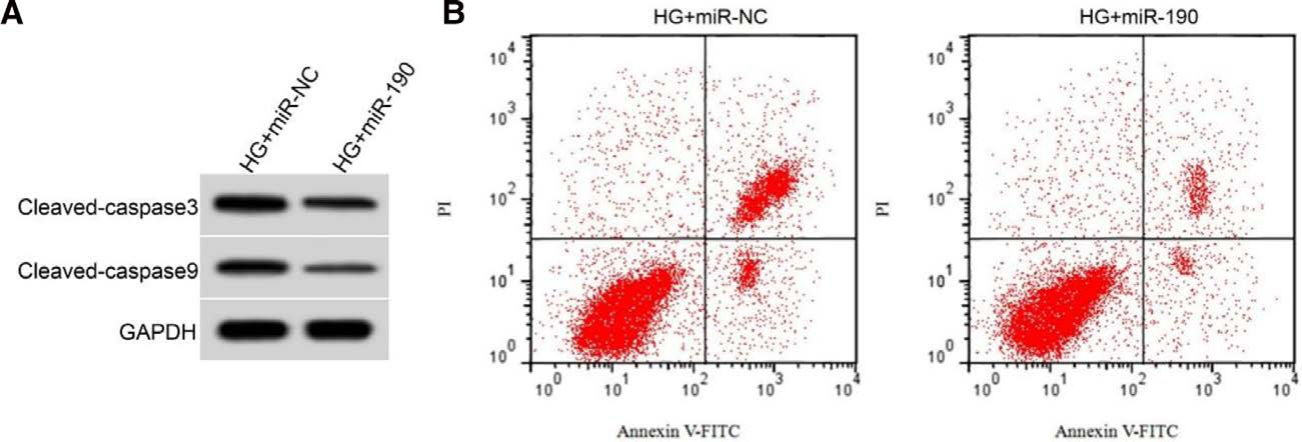
Effect of overexpression of miR-190 on apoptosis of HRECs induced by high glucose. (A) apoptosis-related protein expression, (B) flow diagram of apoptosis. HG = high glucose group.

Compared to the HG + miR-NC group, the HG + miR-190 group showed a significant decrease in apoptosis rate and protein expression of cleaved-caspase3 and cleaved-caspase9 in HRECs (*P* < .05) (Figure 2; Table 5).

**Table 5:** Effect of overexpression of miR-190 on apoptosis of HRECs induced by high glucose (mean ± SD, *n* = 9).

Groups	Apoptosis (%)	Cleaved-caspase 3	Cleaved-caspase 9
HG + miR-NC	32.28 ± 3.03	0.73 ± 0.01	0.59 ± 0.04
HG + miR-190	12.98 ± 1.06*	0.40 ± 0.04*	0.27 ± 0.03*
*t*	18.037	15.461	19.200
*P*	<0.05	<0.05	<0.05

Compared to HG + miR-NC group,

* *P* < .05

### 3.6. Inhibition of miR-190 expression reversed the effect of WEL (40 μmol/L) on high glucose-induced damage in HRECs

**Figure 3.**
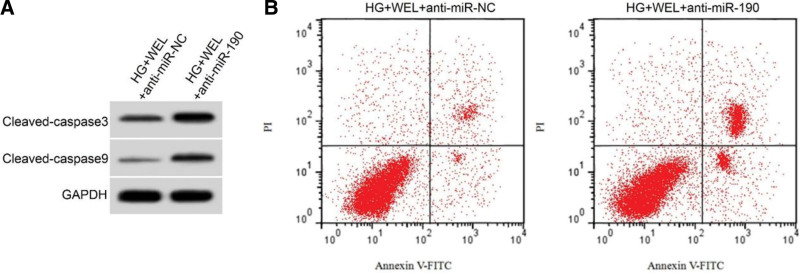
Inhibition of miR-190 expression reversed the effect of wedelactone on the apoptosis of HRECs induced by high glucose. (A) apoptosis-related protein expression, (B) flow diagram of apoptosis. WEL = wedelolactone.

Compared to the HG + WEL + antimiR-NC group, the HG + WEL + antimiR-190 group showed a significant decrease in miR-190 expression and SOD activity in HRECs (*P* < .01), and a significant increase in ROS levels, MDA content, apoptosis rate, and protein expression of cleaved-caspase3 and cleaved-caspase9 (*P* < .01) (Figure 3; Table 6).

**Table 6:** Inhibition of miR-190 expression reversed the effect of wedelactone on high-sugar induced HRECs injury (mean ± SD, *n* = 9).

Groups	miR-190	ROS (%)	SOD (U/mg)	MDA (nmol/mg)	Apoptosis rate (%)	Cleaved-caspase 3	Cleaved-caspase 9
HG + WEL + antimiR-NC	1.00 ± 0.00	130.33 ± 11.38	105.81 ± 10.31	5.01 ± 0.47	9.79 ± 0.87	0.32 ± 0.03	0.15 ± 0.02
HG + WEL + antimiR-190	0.49 ± 0.04*	297.92 ± 23.69*	46.61 ± 4.27*	15.98 ± 1.26*	25.57 ± 2.49*	0.60 ± 0.04*	0.45 ± 0.04*
*t*	38.250	19.130	15.915	24.472	17.948	16.800	20.125
*P*	<0.05	<0.05	<0.05	<0.05	<0.05	<0.05	<0.05

Compared to HG + WEL + miR-NC group,

* *P* < .05

## 4. Discussion

High blood glucose is a major factor leading to retinal damage in the development of DR. Multiple pieces of evidence suggest that excessive production of ROS is associated with high glucose-induced oxidative damage in HRECs. SOD is an antioxidant defense enzyme that directly scavenges ROS to maintain the redox homeostasis. In diabetes, the production of ROS exceeds the clearance capacity, leading to the generation of lipid peroxidation product malondialdehyde (MDA).^[10,11]^ In this study, high glucose stimulation resulted in increased ROS levels, elevated MDA generation, and decreased SOD activity in HRECs, indicating that high glucose stimulation induces oxidative damage in HRECs. WEL has been shown to have broad cellular protective effects.^[12]^ Zhu et al^[13]^ demonstrated that WEL can enhance the antioxidant enzyme activity, reduce pro-inflammatory cytokine levels, inhibit lipid peroxidation reaction, and alleviate inflammation and oxidative damage in foot cells induced by amphotericin B. Results from Ding et al^[14]^ indicated that WEL protects human bronchial epithelial cells from oxidative damage induced by cigarette smoke extract by increasing SOD, catalase (CAT), and glutathione (GSH) activity and reducing MDA content. Additionally, Lu et al^[15]^ reported that WEL alleviates oxidative stress and inflammatory damage in liver tissues induced by carbon tetrachloride by enhancing SOD and GSH-Px activity and reducing the expression of TNF-α, IL-1β, and IL-6. This study demonstrated that WEL inhibits MDA and ROS formation induced by high glucose in a concentration-dependent manner and enhances SOD activity, indicating its protective effect against high glucose-induced damage in HRECs. Oxidative stress is associated with increased apoptosis in HRECs under high glucose conditions. In this study, WEL significantly inhibited high glucose-induced apoptosis in HRECs, reduced the expression levels of apoptosis initiation factor cleaved-caspase9 and apoptosis execution factor cleaved-caspase3, further confirming the ability of WEL to inhibit high glucose-induced damage in HRECs. miR-190 has been shown to be associated with the progression of various diseases, including cancer, Parkinson disease, and myocardial/brain ischemia-reperfusion injury.^[16–18]^ Studies have reported that miR-190 expression is decreased in hepatocellular carcinoma, and upregulation of miR-190 can inhibit liver cancer cell proliferation and metastasis, making it a potential therapeutic target for liver cancer.^[19]^ In a mouse model of Parkinson disease, upregulation of miR-190 can suppress the activation of microglia and inflammatory response, reducing neuronal damage.^[18]^ Moreover, overexpression of miR-190 significantly reduces neurological scores, brain water content, infarct area, and neuronal apoptosis in rats with ischemia-reperfusion injury, protecting against brain ischemia-reperfusion damage.^[17]^ In this study, high glucose stimulation resulted in downregulation of miR-190 expression, while WEL increased miR-190 expression levels in a concentration-dependent manner, suggesting that the protective effect of WEL may be related to the upregulation of miR-190 expression. Functional analysis of miR-190 showed that overexpression of miR-190 can inhibit high glucose-induced oxidative stress damage and cell apoptosis in HRECs, downregulate the protein expression of cleaved-caspase9 and cleaved-caspase3, similar to the protective effect of WEL against high glucose-induced HRECs damage. Furthermore, inhibition of miR-190 expression significantly attenuated the protective effect of WEL against high glucose-induced apoptosis and oxidative stress damage in HRECs, further indicating the protective role of WEL in high glucose-induced HRECs damage through upregulation of miR-190 expression.

In conclusion, WEL can counteract high glucose-induced damage in HRECs by inhibiting oxidative stress and apoptosis, and this mechanism is achieved through upregulation of miR-190 expression, providing preliminary insights into the protective mechanism of WEL in DR and providing experimental evidence for the development of WEL as a therapeutic agent for Dr

## Acknowledgments

We thank all the volunteer who participated in the study.

## Authors contributions

Conceptualization: Xiaojie Cai and Xiaopang Rao.

Data curation: Xiaojie Cai.

Formal analysis: Xiao Wang.

Investigation: Yuping Huang.

Methodology: Yuping Huang.

Project administration: Yuping Huang.

Resource: Xiaopang Rao.

Software: Xiaojie Cai.

Supervision: Xiaopang Rao.

Validation: Xiaojie Cai.

Visualization: Xiaojie Cai.

Writing—original draft: Xiaojie Cai.

Writing—review and editing: Xiaopang Rao.

## References

[1] Martinez B Peplow PV. MicroRNAs as biomarkers of diabetic retinopathy and disease progression. Neural Regen Res. 2019;14:1858–69. 31290435 10.4103/1673-5374.259602 PMC6676865

[2] Lechner J O’Leary OE Stitt AW. The pathology associated with diabetic retinopathy. Vision Res. 2017;139:7–14. 28412095 10.1016/j.visres.2017.04.003

[3] Peng Y-G Zhang L. Wedelolactone suppresses cell proliferation and migration through AKT and AMPK signaling in melanoma. J Dermatolog Treat. 2019;30:389–95. 30252545 10.1080/09546634.2018.1527996

[4] Kučírková T Stiborek M Dúcka M. Anti-cancer effects of wedelolactone: interactions with copper and subcellular localization. Metallomics. 2018;10:1524–31. 30238942 10.1039/c8mt00191j

[5] Nottingham LK Yan CH Yang X. Aberrant IKKα and IKKβ cooperatively activate NF-κB and induce EGFR/AP1 signaling to promote survival and migration of head and neck cancer. Oncogene. 2014;33:1135–47. 23455325 10.1038/onc.2013.49 PMC3926900

[6] Maturi RK Glassman AR Josic K. DRCR Retina Network. Four-year visual outcomes in the protocol w randomized trial of intravitreous aflibercept for prevention of vision-threatening complications of diabetic retinopathy. JAMA. 2023;329:376–85. 36749332 10.1001/jama.2022.25029 PMC10408259

[7] Hershcovici T Wendel CS Fass R. Symptom indexes in refractory gastroesophageal reflux disease: overrated or misunderstood? Clin Gastroenterol Hepatol. 2011;9:816–7. 21791195 10.1016/j.cgh.2011.07.010

[8] Zhai C-L Tang G-M Qian G. miR-190 protects cardiomyocytes from apoptosis induced by H(2)O(2) through targeting MAPK8 and regulating MAPK8/ERK signal pathway. Int J Clin Exp Pathol. 2018;11:2183–92. 31938330 PMC6958226

[9] Li X Yu Z-W Wang Y. MicroRNAs: potential targets in diabetic retinopathy. Horm Metab Res. 2020;52:142–8. 32215885 10.1055/a-1107-2943

[10] Liu L Xu H Zhao H. STEAP4 inhibits HIF-1α/PKM2 signaling and reduces high glucose-induced apoptosis of retinal vascular endothelial cells. Diabetes Metab Syndr Obes. 2020;13:2573–82. 32765036 10.2147/DMSO.S251663 PMC7381765

[11] Li Q Pang L Shi H. High glucose concentration induces retinal endothelial cell apoptosis by activating p53 signaling pathway. Int J Clin Exp Pathol. 2018;11:2401–7. 31938352 PMC6958250

[12] Yan T Yang S Zhou X. Chronic kidney disease among greenhouse workers and field workers in China. Chemosphere. 2022;302:1–7. 10.1016/j.chemosphere.2022.134905 35561762

[13] Zhu M-M Wang L Yang D. Wedelolactone alleviates doxorubicin-induced inflammation and oxidative stress damage of podocytes by IκK/IκB/NF-κB pathway. Biomed Pharmacother. 2019;117:109088. 31202173 10.1016/j.biopha.2019.109088

[14] Ding S Hou X Yuan J. Wedelolactone protects human bronchial epithelial cell injury against cigarette smoke extract-induced oxidant stress and inflammation responses through Nrf2 pathway. Int Immunopharmacol. 2015;29:648–55. 26411738 10.1016/j.intimp.2015.09.015

[15] Lu Y Hu D Ma S. Protective effect of wedelolactone against CCl4-induced acute liver injury in mice. Int Immunopharmacol. 2016;34:44–52. 26921731 10.1016/j.intimp.2016.02.003

[16] Sun Q Wang S Chen J. MicroRNA-190 alleviates neuronal damage and inhibits neuroinflammation via Nlrp3 in MPTP-induced Parkinson’s disease mouse model. J Cell Physiol. 2019;234:23379–87. 31232472 10.1002/jcp.28907

[17] Yu Y Cao X-C. miR-190-5p in human diseases. Cancer Cell Int. 2019;19:257. 31624470 10.1186/s12935-019-0984-x PMC6781386

[18] Jiang C Dong N Feng J. MiRNA-190 exerts neuroprotective effects against ischemic stroke through Rho/Rho-kinase pathway. Pflugers Arch. 2021;473:121–30. 33196911 10.1007/s00424-020-02490-2

[19] Xiong Y Wu S Yu H. miR-190 promotes HCC proliferation and metastasis by targeting PHLPP1. Exp Cell Res. 2018;371:185–95. 30092222 10.1016/j.yexcr.2018.08.008

